# Preparation, Characterization and Biological Applications of Biosynthesized Silver Nanoparticles with Chitosan-Fucoidan Coating

**DOI:** 10.3390/molecules23061429

**Published:** 2018-06-12

**Authors:** Jayachandran Venkatesan, Sandeep Kumar Singh, Sukumaran Anil, Se-Kwon Kim, Min Suk Shim

**Affiliations:** 1 Yenepoya Research Center, Yenepoya (Deemed to Be University), Deralakatte, Mangalore 575018, Karnataka, India; venkatjchem@gmail.com; 2 Division of Bioengineering, Incheon National University, Incheon 22012, Korea; 3 Department of Pharmaceutical Sciences and Technology, Birla Institute of Technology, Mesra, Ranchi 835215, Jharkhand, India; sandympharm@yahoo.com; 4 Marine Bioprocess Research Centre and Department of Marine Bio-Convergence Science, Pukyong National University, Sinseon-ro 365, Nam-gu, Busan 608739, Korea; 5 Department of Periodontics, Saveetha Dental College and Hospitals, Saveetha University, Poonamallee High Road, Chennai 600077, India; drsanil@gmail.com

**Keywords:** chitosan, fucoidan, silver nanoparticles, antimicrobial activity

## Abstract

Silver nanoparticles (AgNPs) are gaining a great deal of attention in biomedical applications due to their unique physicochemical properties. In this study, green synthesis of AgNPs was developed using seaweed polysaccharide fucoidan. The AgNPs were further coated with chitosan to form an electrolyte complex on the surface. The developed chitosan–fucoidan complex-coated AgNPs were characterized using UV-visible spectroscopy, Fourier transform infrared spectroscopy (FT-IR), and transmission electron microscopy (TEM). FT-IR results suggested strong polyelectrolyte complexation between fucoidan and chitosan. The developed chitosan–fucoidan complex-coated AgNPs significantly inhibited microbial growth. Moreover, the AgNPs showed efficient anticancer activity in human cervical cancer cells (HeLa). This study demonstrated that chitosan–fucoidan complex-coated AgNPs hold high potential for food and cosmeceutical applications.

## 1. Introduction

Nanotechnology is gaining much attention in several fields, such as energy, medicine, and environmental areas [1,2,3]. Engineered metal nanoparticles have attracted widespread interest in the fields of catalysis, photonics, photography, regenerative medicine, drug delivery, gene therapy and biosensors due to their unique properties [4,5,6]. Engineered metal nanoparticles can be produced in multiple ways, such as chemical synthesis [5] and biosynthesis methods [7]. Chemical synthesis has advantages in controlling the size of metal nanoparticles during the synthetic procedure [5]. However, some toxic chemicals are often involved during the preparation process [8]. Alternatively, biosynthesis of metal nanoparticles using biocompatible microbes and plants can avoid toxicity issues [9]. Biosynthesized silver nanoparticles (AgNPs) have been intensively developed in recent years due to their excellent antimicrobial properties [10,11,12]. Biosynthesis of AgNPs can be obtained through reducing silver ions into AgNPs by microbes and plants. Biosynthesis of AgNPs using marine-derived polysaccharides has been attempted previously [13]. For example, Leung et al. showed that AgNPs could be prepared using carboxymethylated-curdlan and fucoidan to reduce silver nitrate ions [13,14]. Wei et al. and Murugadoss et al. developed AgNPs with chitosan [10]. These polysaccharides were not only utilized as reducing agents to reduce silver ions to AgNPs, but also as stabilizing agents. 

Fucoidan is an anionic polysaccharide commonly isolated from brown seaweeds. Fucoidan has several biological activities, including anticancer, anticoagulant, antioxidant, and anti-inflammatory activities [15,16,17,18,19,20,21,22,23]. Chitosan is a cationic polysaccharide derived from chitin. Chitin is considered the second most abundant polysaccharide next to the cellulose [24,25]. Chitosan has been extensively studied for various biomedical applications, including wound healing, tissue engineering, and drug delivery, due to its biocompatibility and biodegradability [26,27,28,29,30]. Chitosan and fucoidan are oppositely charged polysaccharides, thus enabling the formation of polyelectrolyte complexes via self-assembly [31,32,33]. Several studies have suggested that chitosan–fucoidan polyelectrolyte/nanoparticles have great advantages in drug delivery [31,32,33,34,35,36,37,38,39] and tissue engineering [40,41,42] due to their efficient drug encapsulation and cellular uptake. 

In this study, chitosan–fucoidan complex-coated AgNPs were synthesized and utilized for antimicrobial and anticancer activities. The chitosan–fucoidan-coated AgNPs were characterized by Fourier transform infrared spectroscopy (FT-IR), dynamic light scattering (DLS), and transmission electron microscopy (TEM) analysis. Antibacterial and anticancer activities of chitosan–fucoidan complex-coated AgNPs were also studied.

## 2. Results and Discussion

### 2.1. Preparation of Chitosan–Fucoidan Complex-Coated AgNPs

**Figure 1:**
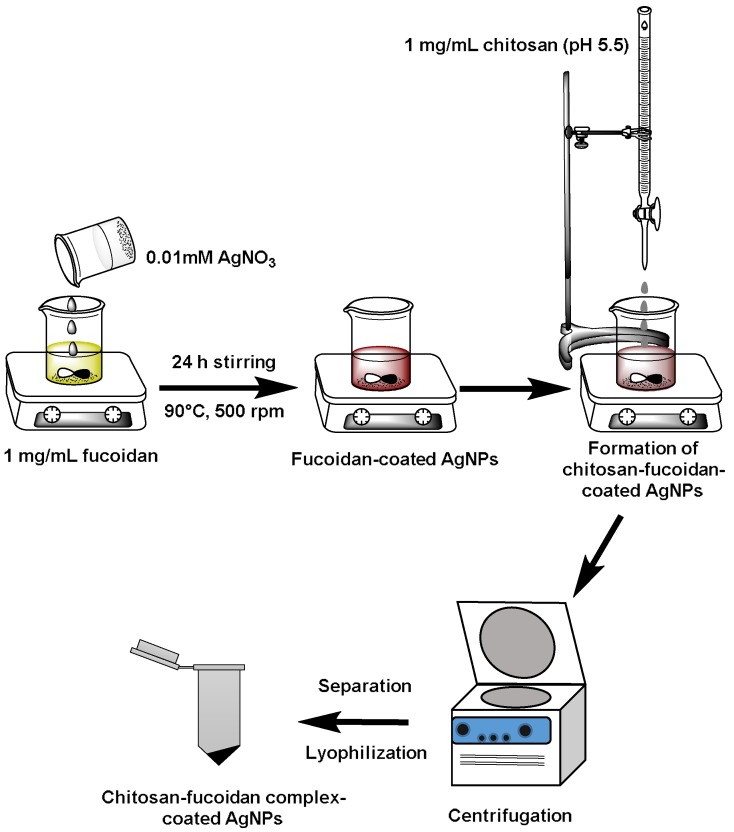
Schematic representation for the preparation of chitosan–fucoidan complex-coated silver nanoparticles (AgNPs).

Figure 1 shows the schematic for the fabrication of chitosan–fucoidan-coated AgNPs. First, fucoidan was mixed with silver nitrate (AgNO_3_) solution to form AgNPs. Fucoidan was used as capping agents as well as reducing agents for the formation of stable AgNPs [43]. Then, chitosan was added to the fucoidan-coated AgNPs to form stable AgNPs coated with chitosan–fucoidan polyelectrolyte complexes. Centrifugation was used to remove the residual AgNO_3_, free chitosan, and fucoidan from the chitosan–fucoidan complex-coated AgNPs. The final product was obtained by lyophilization. 

### 2.2. UV-Visible (UV-Vis) Spectroscopy

**Figure 2:**
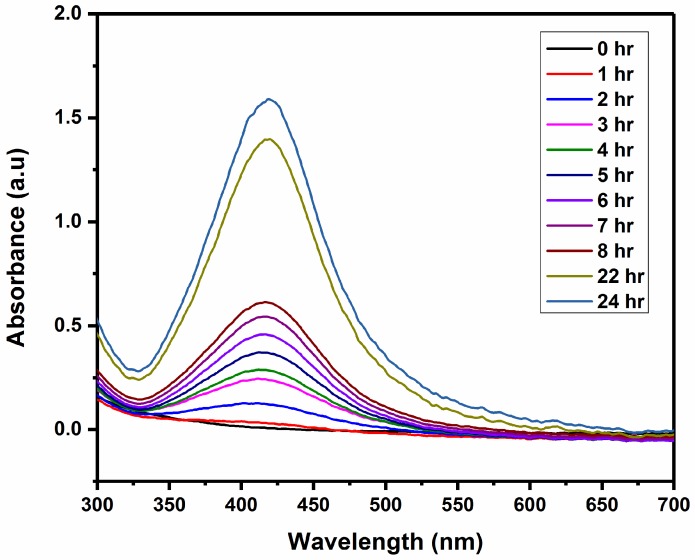
UV-visible (UV-Vis) spectrum of AgNPs synthesized with fucoidan at different reaction time.

In a previous study, AgNPs were prepared by using fucan of *Spatoglossum schröederi* brown seaweed [44]. According to that study, fucan reduces the silver ions to form the stable fucan-coated AgNPs [44]. In this study, *Fucus vesiculosus* brown seaweed-derived fucoidan was utilized to prepare AgNPs. Fucoidan from *Fucus vesiculosus* has been utilized for anticancer therapy [45]. UV-Vis spectroscopy of fucoidan-coated nanoparticles was obtained at different time intervals. Figure 2 shows the UV-Vis spectrum of biosynthesized AgNPs at different time intervals. As shown in Figure 2, during the first hour, AgNPs were not formed. However, surface plasmon resonance (SPR) peak of AgNPs at 418 nm was observed after 2 h of reaction, and the intensity increased with time. The maximum intensity of the SPR peak was observed after 24 h of interaction of fucoidan with silver ions. 

The SPR peak of AgNPs at 418 nm was consistent with the results reported by other groups [37]. They developed biosynthesized AgNPs with water extracts of *Sargassum muticum* [46]. 

### 2.3. Particle Size Analysis Using DLS 

**Figure 3:**
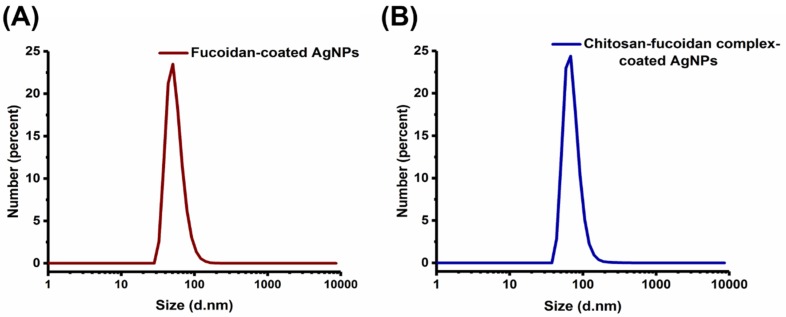
Size of (**A**) fucoidan-coated AgNPs and (**B**) chitosan–fucoidan complex-coated AgNPs.

The size of fucoidan-coated AgNPs and chitosan–fucoidan complex-coated AgNPs were measured by DLS (Figure 3A,B). The size and polydispersity index (PDI) of the fucoidan-capped AgNPs and chitosan–fucoidan complex-coated AgNPs were found to be 53.19 ± 2.23 nm (PDI: 0.39 ± 0.04) and 73.09 ± 9.54 nm (PDI: 0.47 ± 0.05), respectively. DLS results shown in Figure 3A,B indicate that the average size of chitosan–fucoidan complex-coated AgNPs (73.1 nm) is larger than that of fucoidan-coated AgNPs (53.1 nm). This result implies that cationic chitosan is electrostatically bound to the surface of anionic fucoidan-coated AgNPs, resulting in increased particle size. Importantly, there was no severe aggregation of particles when the fucoidan-coated AgNPs were complexed with chitosan, indicating their high colloidal stability. 

Leung et al. reported the size of fucoidan-coated AgNPs as approximately 40–80 nm [13]. This result is in good agreement with our data. In another study, Amorim et al. reported the size of fucan-coated AgNPs as 210.44 ± 29.13 nm [44]. It has clearly been found that the size of AgNPs mainly depends on the molecular weights of fucoidan. The zeta potentials of fucoidan-coated AgNPs and chitosan–fucoidan complex-coated AgNPs were −36.9 ± 0.91 mV and −32.1 ± 0.95 mV, respectively. The negative charge of the AgNPs indicated the presence of sulfate groups on the surface of the AgNPs. There are several reports suggesting that negative charged polysaccharides usually produce AgNPs with negative surface charges [47,48]. The increased zeta-potential of chitosan–fucoidan complex-coated AgNPs can be attributed to the presence of positively charged chitosan on the AgNPs. 

### 2.4. Fourier Transform Infrared (FT-IR) Spectroscopy

**Figure 4:**
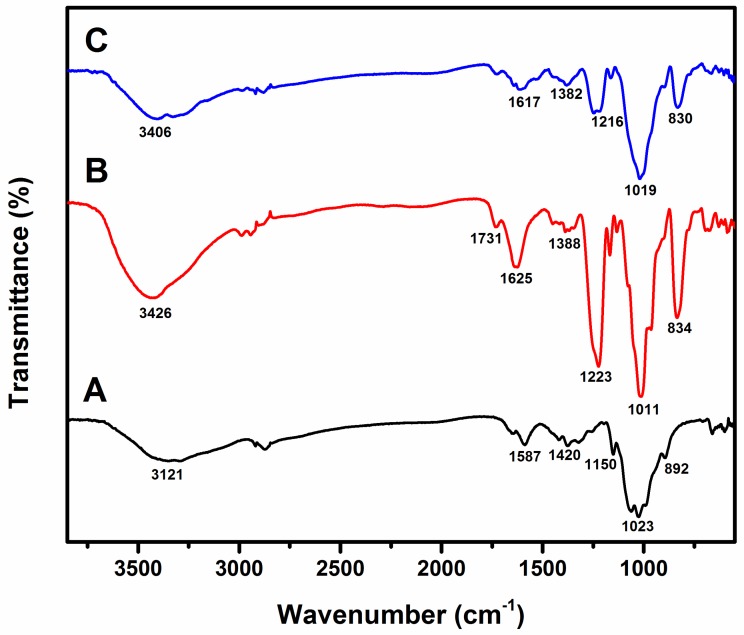
Fourier transform infrared spectroscopy (FT-IR) spectrum of (**A**) chitosan, (**B**) fucoidan, and (**C**) fucoidan-coated AgNPs with chitosan.

Figure 4 shows the FT-IR spectrum of (A) chitosan, (B) fucoidan, and (C) chitosan–fucoidan complex-coated AgNPs. The characteristic peaks of chitosan were observed in Figure 4A, which are 892 cm^−1^, 1023 cm^−1^, 1150 cm^−1^, 1420 cm^−1^, 1587 cm^−1^, and 3121 cm^−1^. The peak at 892 cm^−1^ indicates the C–O–C bridge as well as glycosidic linkage. The peak at 1023 cm^−1^ shows the presence of skeletal vibration involving C–O–C [49].

Figure 4B shows the characteristic peaks of fucoidan at 834 cm^−1^, 1011 cm^−1^, 1223 cm^−1^, 1388 cm^−1^, 1625 cm^−1^, 1731 cm^−1^, and 3426 cm^−1^. The peaks at 1011 cm^−1^, 1223 cm^−1^, and 834 cm^−1^ correspond to the S=O and C–O–S stretching of the sulfate groups in fucoidan [35]. Figure 4C represents the FT-IR spectrum of chitosan–fucoidan complex-coated AgNPs. The characteristic peaks are 830 cm^-1^, 1019 cm^−1^, 1216 cm^−1^, 1382 cm^−1^, 1617 cm^−1^, and 3406 cm^−1^. The peaks at 830 indicate the presence of the sulfate groups of fucoidan, whereas the peaks at 1019 cm^−1^ and 1216 cm^−1^ correspond to the chitosan moieties. The FT-IR spectra clearly confirm the formation of chitosan–fucoidan polyelectrolyte complexation. There are no detectable peaks for AgNPs due to the trace amount of AgNPs. 

### 2.5. Morphology of AgNPs 

**Figure 5:**
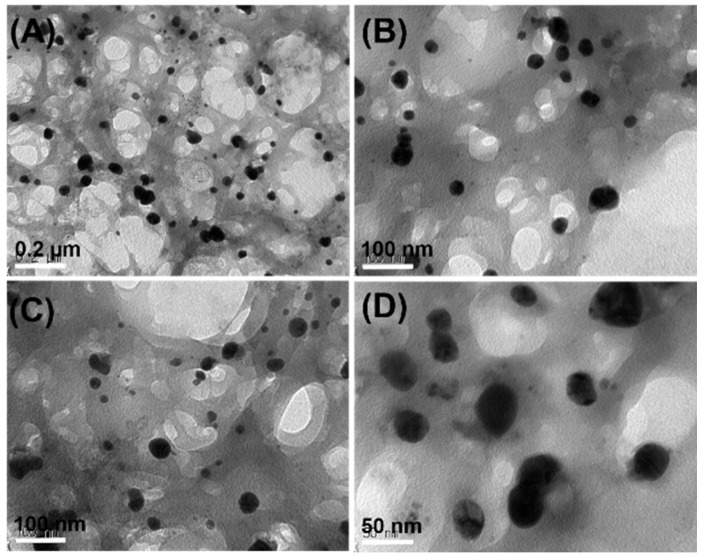
Transmission electron microscopy (TEM) images of chitosan–fucoidan complex-coated AgNPs at different magnifications (**A**) 0.2 µm, (**B**,**C**) 100 nm, and (**D**) 50 nm.

Figure 5A–D shows the size and morphology of AgNPs coated with chitosan and fucoidan at different magnifications. The TEM results indicate that AgNPs show spherical shapes, but not in uniform size. The average size of the chitosan–fucoidan complex-coated AgNPs was around 50 nm. The size of the chitosan–fucoidan complex-coated AgNPs determined by TEM was slightly smaller than that of the chitosan–fucoidan complex-coated AgNPs determined by DLS (Figure 3A,B). The DLS results provide hydrodynamic diameters of the chitosan–fucoidan complex-coated AgNPs dispersed in water, while TEM results provide the actual size of the dried AgNPs. Therefore, the size of the chitosan–fucoidan complex-coated AgNPs determined by DLS should be smaller than that of the AgNPs determined by TEM. 

### 2.6. Antibacterial Activity of AgNPs

**Figure 6:**
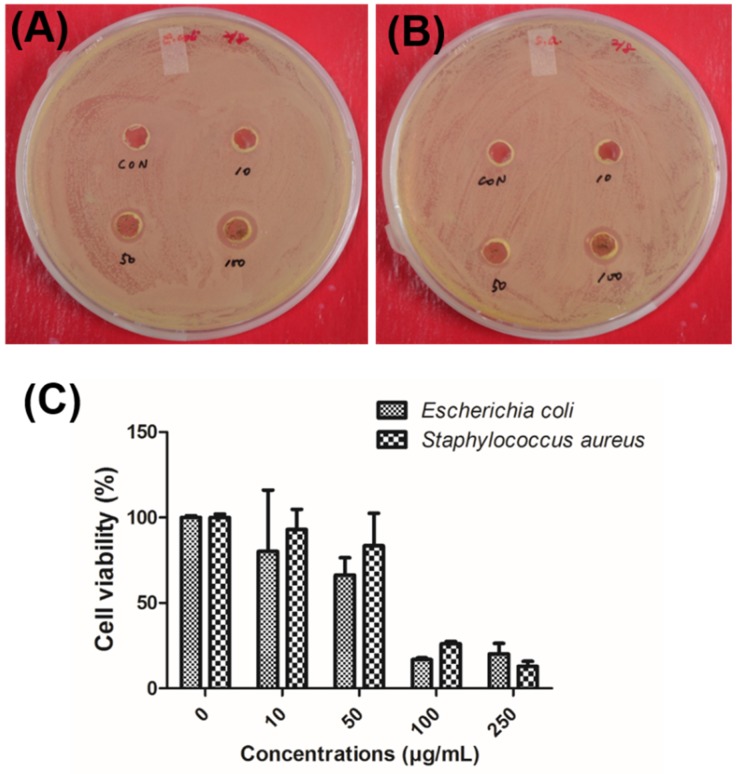
Antimicrobial activity of chitosan–fucoidan complex-coated AgNPs at different concentrations (10 µg, 50 µg, and 100 µg). (**A**) *E. coli* colonies. (**B**) *S. aureus* colonies. *(***C**) Viability of *E. coli and S. aureus* after treated with chitosan–fucoidan complex-coated AgNPs.

The antimicrobial activities of chitosan–fucoidan complex-coated AgNPs were evaluated against Gram-positive *Staphylococcus aureus* (*S. aureus*) and Gram-negative *Escherichia coli* (*E. coli*) using an agar well diffusion method. After both bacteria were incubated with the AgNPs, clear zones were observed against all the tested organisms and were recorded in circle size. The chitosan–fucoidan complex-coated AgNPs showed inhibition of growth in both *E. coli* (Figure 6A) and *S. aureus* (Figure 6B). Furthermore, as expected, no inhibition was detected when using the negative control media. As shown in Figure 6A,B, chitosan–fucoidan complex-coated AgNPs revealed concentration-dependent antibacterial activity. The highest growth inhibition in *E. coli* (3 ± 0.3 mm) was observed when incubated at 100 µg/mL of chitosan–fucoidan complex-coated AgNPs. Likewise, *S. aureus* colonies treated with 100 µg/mL of chitosan–fucoidan complex-coated AgNPs exhibited the highest growth inhibition (2 ± 0.2 mm). 

In addition, the antimicrobial activities of chitosan–fucoidan complex-coated AgNPs were evaluated against *E. coli* and *S. aureus* using an MTT assay (Figure 6C). The IC_50_ values of the chitosan–fucoidan complex-coated AgNPs on the tested microorganisms were 61.3 µg/mL against *E. coli* and 70 µg/mL against *S. aureus*. *E. coli* was more sensitive than *S. aureus* in the same concentration. This study indicates that chitosan–fucoidan complex-coated AgNPs have excellent biocidal effects, showing potential in reducing bacterial growth in practical applications.

### 2.7. Anticancer Activity of Chitosan–Fucoidan Complex-Coated AgNPs

**Figure 7:**
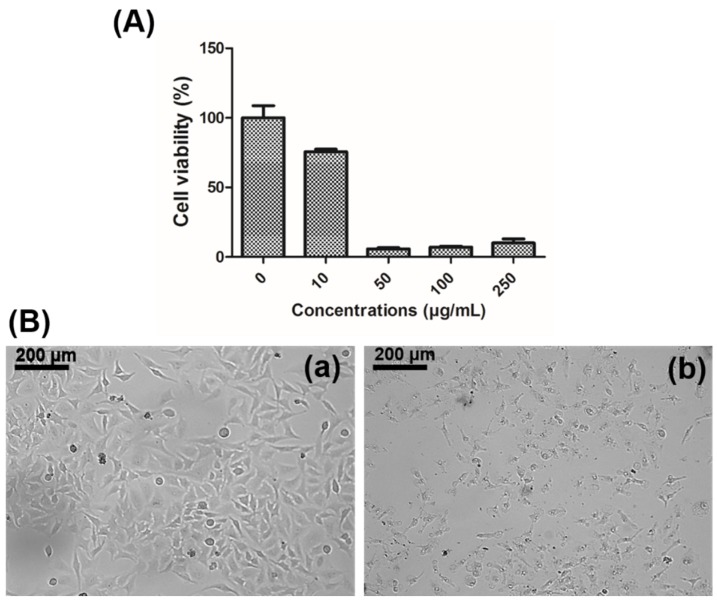
(**A**) Cytotoxicity effects of chitosan–fucoidan complex-coated AgNPs in human cervical cancer cells (HeLa) at different concentrations. (**B**) Optical microscopy images of HeLa cells treated with (**a**) Blank and (**b**) 250 µg/mL of chitosan–fucoidan complex-coated AgNPs.

The cytotoxicity of chitosan–fucoidan complex-coated AgNPs was assessed against human cervical cancer cells (HeLa). The cells were treated with different concentrations of AgNPs (10, 50, 100, and 250 µg/mL) for 24 h. Significant cytotoxicity was observed at the 50 µg/mL of concentration, and the IC_50_ value was found to be around 35 µg/mL (Figure 7A). Jang et al. reported that biosynthesized AgNPs have efficient anticancer activity by upregulation of p53 gene expression [50]. The cytotoxicity in cancer cells is associated with changes in morphology and loss of adherence and rounding, which was clearly shown in Figure 7B [49].

### 2.8. Flow Cytometry Analysis

**Figure 8:**
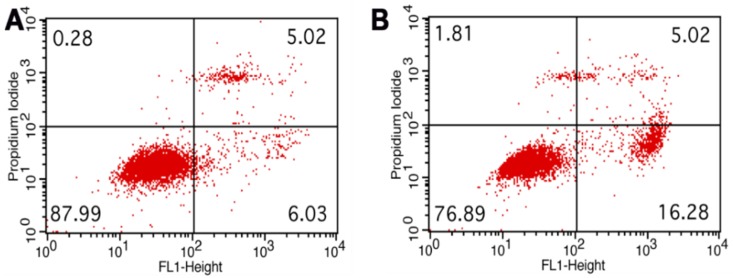
Flow cytometry analysis of (**A**) untreated HeLa cells and (**B**) HeLa cells treated with chitosan–fucoidan complex-coated AgNPs at 250 µg/mL.

Figure 8 shows the flow cytometry analysis of untreated HeLa cells and HeLa cells treated with chitosan–fucoidan complex-coated AgNPs (250 µg/mL). As shown in Figure 8A, 6.03% of cells were located in the lower right quadrant indicating early apoptosis of cells. As shown in Figure 8B, the cell population in the lower left quadrant was 16.28%, indicating the increased apoptosis by chitosan–fucoidan complex-coated AgNPs. 

## 3. Materials and Methods

Fucoidan, chitosan, and AgNO_3_ were purchased form Sigma Aldrich (St. Louis, MO, USA). HeLa cells were purchased from American Type Culture Collection (ATCC) (Manassas, VA, USA). Dulbecco’s modified eagle medium (DMEM) and fetal bovine serum (FBS) were obtained from Lonza Chemicals (Walkersville, MD, USA). [3-(4,5-dimethylthiazol-2-yl)-2,5-diphenyltetrazolium bromide] (MTT) was purchased from Sigma Aldrich (St. Louis, MO, USA). Apoptosis kit (FITC Annexin V Apoptosis Detection Kit) was purchased from Biosciences (Heidelberg, Germany).

### 3.1. Preparation of Fucoidan-Coated AgNPs

First, fucoidan (1 mg/mL) was dissolved in water and taken in a closed container. Then, 0.01 mM AgNO_3_ was added into the fucoidan solution and heated on a hot plate at 90 °C. Further, the solution was continuously stirred with a magnetic stirrer for 24 h to form AgNPs. After that, 2 mL of chitosan (1 mg/mL, pH: 5.5) was added to 8 mL of fucoidan-coated AgNPs solution to form chitosan–fucoidan complex-coated AgNPs. The AgNPs were separated from the solution by centrifugation at 13,000 rpm for 20 min. The supernatant was discarded, and the pellet was dispersed in water and centrifuged again. The AgNPs were dispersed in water and freezed at −24 °C overnight, followed by lyophilization. 

### 3.2. UV-Visible Spectroscopy

Reduction of silver ions by fucoidan was monitored using UV-visible spectroscopy (GeneQuant 1300, GE Healthcare, Piscataway, NJ, USA). An aliquot of the reaction mixture was collected periodically and scanned using a spectrophotometer at wavelengths between 200 and 800 nm with a resolution of 1 nm.

### 3.3. DLS Analysis

Size distribution of the AgNPs was characterized by dynamic light scattering using a Malvern Zetasizer Nano ZS (Worcestershire, UK).

### 3.4. Fourier Transform Infrared Spectroscopy

FT-IR analysis (Scientific Instruments LLC, Madison, WI, USA) was performed to determine the chemical interaction between chitosan and fucoidan. A Nicolet iS10 Thermo Electron Spectrometer was used in this study. The ATR method was used in this study. 

### 3.5. Transmission Electron Microscopy Analysis

Surface morphologies and nanoparticle size of AgNPs were obtained using a transmission electron microscope (H7500, Hitachi Ltd., Tokyo, Japan) at 120 kV. 

### 3.6. Antimicrobial Activity Test of AgNPs

Two different bacteria (*E. coli* and *S. aureus*) were used in this study. All strains were maintained in Luria Broth (LB) agar (10 g/L sodium chloride, 5 g/L yeast extract, 10 g/L trypton, 15 g/L agar) as stock cultures at 4 °C, as reported in a previous study by Kalimuthu et al. [51]. The antimicrobial activities of chitosan–fucoidan complex-coated AgNPs were determined by an agar plate well diffusion method [52]. Bacteria were cultured in LB broth; 200 μL of an initial inoculum (0.2 × 10^8^ CFU) of each strain in the LB agar media was applied and uniformly spread in the well. Subsequently, 100 μL of chitosan–fucoidan complex-coated AgNPs solutions at 10, 50, and 100 μg/mL concentrations were added into 5 mm diameter wells and incubated for 24 h at 37 °C. LB broth (100 μL) was used as a negative control. After incubation, the zones of inhibition were measured as per the previously reported method [53]. 

### 3.7. Cytotoxicity Assay

MTT assay was used to estimate the antibacterial activity of chitosan–fucoidan complex-coated AgNPs. The different concentrations (10, 50, and 100 µg/mL) of the nanoparticles were added to the well containing 500 µL of diluted bacterial culture (1 × 10^6^ CFU/mL) and kept in a shaking incubator at 37 °C for 24 h. MTT assay was performed according to the previous study [54]. IC_50_ value was measured by plotting the viability with concentration.

### 3.8. Cytotoxicity Effects of Nanoparticles in Cancer Cells 

Cytotoxicity effects of the developed chitosan-fucoidan complex-coated AgNPs were checked with HeLa cells according to our previous study [11]. In this study, different concentrations (10, 50, 100 and 250 μg/mL) of nanoparticles were used. 

### 3.9. Optical Microscopy Analysis 

HeLa cells were cultured in 24-well plates at a density of 1 × 10^4^ cells per well. After 24 h of incubation, cells were treated with chitosan–fucoidan complex-coated AgNPs. After incubation for 24 h, morphologies of the cells were observed using an optical microscope (CTR 6000, Leica, Wetzlar, Germany).

### 3.10. Apoptosis Analysis

Apoptotic cell death of HeLa cells induced by chitosan–fucoidan complex-coated AgNPs was measured by flow cytometry analysis after Annexin V and PI staining. The flow cytometry analysis was performed according to our previous study [11]. HeLa cells were placed in 6-well culture plate for 24 h at 37 °C, and cells were treated with 250 µg/mL of chitosan–fucoidan complex-coated AgNPs. Further, cells were incubated for 6 h, washed with PBS, trypsinized, and collected. Then, the cells were resuspended in Annexin V binding buffer solution. The cells were stained according to the manufacturer’s protocol (BD Biosciences). Fluorescent intensities of Annexin V and PI were measured using a flow cytometer (FACS Calibur, BD Biosciences, Heidelberg, Germany). 

### 3.11. Statistical Analysis

All the experiments were performed in triplicate. Data were analyzed using Student’s *t*-test at a significance level of *p* < 0.01 and presented as mean ± standard deviation.

## 4. Conclusions

Biosynthesis of AgNPs was achieved by using fucoidan as reducing and capping agents. The formation of AgNPs was confirmed by UV-Vis spectroscopy. The AgNPs were further coated with chitosan to form chitosan–fucoidan complex-coated AgNPs. The particle size and polydispersity index of the fucoidan-coated AgNPs and chitosan–fucoidan complex-coated AgNPs were 53.19 ± 2.23 nm and 73.09 ± 9.54 nm, respectively. Electrostatic interaction was observed between the chitosan and fucoidan, which was confirmed by FT-IR spectroscopy. The developed chitosan–fucoidan complex-coated AgNPs significantly inhibited the growth of *E. coli* and *S. aureus*. The anticancer activity of the AgNPs was also investigated with HeLa cells. The IC_50_ value of the AgNPs was 35 µg/mL. This study suggests that chitosan–fucoidan complex-coated AgNPs are promising candidates for food and cosmeceutical applications.
